# Hazardous alcohol consumption among older adults: A comprehensive and multi-national analysis of predictive factors in 13,351 individuals

**DOI:** 10.1192/j.eurpsy.2020.112

**Published:** 2020-12-21

**Authors:** Stephan Listabarth, Benjamin Vyssoki, Thomas Waldhoer, Andrea Gmeiner, Sandra Vyssoki, Andreas Wippel, Victor Blüml, Maria Gruber, Daniel König

**Affiliations:** 1 Clinical Division of Social Psychiatry, Department of Psychiatry and Psychotherapy, Medical University of Vienna, Vienna, Austria; 2 Center for Public Health, Department of Epidemiology, Medical University of Vienna, Vienna, Austria; 3 Department of Health Sciences, St. Pölten University of Applied Sciences, Sankt Pölten, Austria; 4 Department of Psychoanalysis and Psychotherapy, Medical University of Vienna, Vienna, Austria

**Keywords:** Addiction, aged, alcohol use disorder, epidemiology, personality

## Abstract

**Background:**

Older adults exhibit heightened vulnerability for alcohol-related health impairments. Increases in the proportion of older adults within the European Union’s total population and prevalence rates of alcohol use disorders in this age group are being observed. This large scale international study was conducted to identify those older adults with an increased risk to engage in hazardous drinking behaviour.

**Methods:**

Socio-demographic, socio-economic, personality characteristics (Big Five Inventory, *BFI-10*), and alcohol consumption patterns of 13,351 individuals from 12 different European countries, collected by the Survey of Health, Aging, and Retirement in Europe, were analyzed using regression models.

**Results:**

Age, nationality, years of education, as well as personality traits, were significantly associated with alcohol intake. For males, extraversion predicted increased alcohol intake (RR = 1.11, CI = 1.07–1.16), whereas conscientiousness (RR = 0.93, CI = 0.89–0.97), and agreeableness (RR = 0.94, CI = 0.90–0.99), were associated with a reduction. For females, openness to new experiences (RR = 1.11, CI = 1.04–1.18) predicted increased alcohol intake. Concerning excessive drinking, personality traits, nationality, and age-predicted consumption patterns for both sexes: Extraversion was identified as a risk factor for excessive drinking (OR = 1.15; CI = 1.09–1.21), whereas conscientiousness was identified as a protective factor (OR = 0.87; CI = 0.823–0.93).

**Conclusion:**

Hazardous alcohol consumption in the elderly was associated with specific personality characteristics. Preventative measures, crucial in reducing deleterious health consequences, should focus on translating the knowledge of the association of certain personality traits and alcohol consumption into improved prevention and treatment.

## Introduction

The proportion of older adults (≥50 years of age) as well as senior adults (≥65 years of age) among the European Union’s total population has been rapidly growing in recent decades, and demographic projections show this trend to continue in the future. According to the European Commission, the share of people aged 65 or older is estimated to have reached 30% by 2070 (up from about 20% today) [1]. These developments highlight the increasing importance of preventive measures as well as therapeutic and diagnostic measures focusing on the mental and physical health care of older adults. One of the challenges is the growing relevance of hazardous alcohol consumption within the aging population. Previous epidemiological studies have shown robust evidence for an upward tendency in the prevalence of alcohol use disorders (AUD) and other substance abuse disorders (SUD) in older adults [2–5], causing intensifying strain on health care systems [2]. Importantly, older individuals are more vulnerable to alcohol-related damage, as (a) the metabolic capacity to deal with toxic agents is often reduced [6]; (b) due to a higher amount of prescribed drugs, elderly are at a higher risk for pharmacological interactions [3,7]; and therefore, (c) levels of harmful alcohol consumption are likely to be exceeded more often. The negative consequences of frequent and excessive alcohol consumption are a global phenomenon, accounting for a significant burden on the health of individuals affected as well as the society and the health system overall [8]. Globally, more than 3 million deaths (5.3% of all deaths) per year [9] and over 5% of all disability-adjusted-life-years [10] are postulated to be associated with harmful alcohol consumption or related secondary diseases. However, the ability to determine precise epidemiological numbers related to alcohol consumption and *AUD* is limited, potentially underestimating the prevalence of these conditions [11–13].

It is known from previous studies that individual alcohol consumption patterns are influenced by specific factors, including socio-economic, socio-demographic, biographical characteristics (e.g., stressful life events), and personality traits [3,14–17]. However, research on potential risk factors for alcohol consumption within the elderly population is scarce. Furthermore, the influence of personality characteristics on alcohol consumption has either been researched in North America (mainly within college students) or in other single-country studies [18–22], but not within the highly relevant group of older adults in several European countries.

Therefore, this study aims to comprehensively examine factors, with a focus on individual personality characteristics, which modify alcohol consumption behavior by analyzing a large-scale multi-national study of the older adult population.

Besides the scientific interest, there are also relevant clinical implications drawn from the findings of this study. First, it could help to better target those individuals at higher risk for hazardous alcohol consumption. Second, some of those factors, such as personality characteristics, are also highly relevant for prognosis, the course of disease, and the risk of relapse. Furthermore, they might also be valuable indicators for selecting the most feasible type of treatment for every patient on an individual level. This is why further efforts in investigating specific risk factors, trajectories, and treatment modifying *AUD* characteristics in this patient collective are of highest interest.

## Methods

### Data

#### Study population

Data were extracted from the Survey of Health, Aging, and Retirement in Europe (*SHARE*; SHARE, Wave 7, DOI: 10.6103/SHARE.w7.700) [23]. SHARE is a database of individual data on health, socio-economic status, and social/family networks of elderly (50 years of age or older) and is periodically conducted in 27 European countries and Israel. Data collection for the seventh (and most recent) wave of this survey took place in 2017 and 2018 and was performed using computer-assisted personal interviewing (*CAPI*; face-to-face interviews conducted by support of a laptop application; also see Börsch-Supan et al. [24] for methodological details).

#### Assessments

By use of the *CAPI* method, specific socio-demographic and socio-economic parameters (e.g., age, household income, occupation, educational obtainment) were surveyed. Furthermore, among other validated psychometric scales, the Big Five Inventory (*BFI-10*), was applied to assess the five-factor model of personality. This is one of the most comprehensive and best-established models to assess personality [4,5]. It aims to empirically describe personality along five major dimensions: neuroticism, extraversion, openness, agreeableness, and conscientiousness [4,5]. These five personality dimensions show clear heritable characteristics [6,7], have been found to be associated with several psychiatric disorders [8,9,10] and have frequently been used to investigate the complex interplay of *SUD* and personality within the last decades [20,25–32]. Data have shown mixed-sex samples to exhibit lower effect sizes than single-sex samples, indicating that mixing sexes in data analysis may obscure effects [32]. The *BFI-10* evaluates the five major dimensions of personality on a 5-level rating scale and was developed as a derivative of the Big Five Inventory (*BFI-44*), especially for research projects with limited time resources [33]. The *BFI-44* consists of 44 items and is the most commonly used. However, the shorter *BFI-10* also achieved sufficient levels of validity and reliability: Discriminant and structural validity had been shown to be equivalent with those of the *BFI-44* and also reliability yielded satisfying results by capturing 70% of the variance and retaining 85% of the test–retest reliability of the *BFI-44* [33].

As one of many indicators concerning behavioral risk, participants of the *SHARE* survey were asked for the number of alcoholic drinks consumed during the last 7 days and the frequency of excessive drinking, that is, consuming six or more alcoholic drinks per occasion during the last 3 months. An alcoholic drink was defined as 10 g pure alcohol, corresponding to approximately a can of beer (33 cl), a glass of wine (12 cl), or a shot glass of spirituous (4 cl). While the measurement “alcoholic drink” corresponds roughly to a “standard drink,” no agreement between countries in Europe exists on the amount of alcohol contained within a standard drink (amount of alcohol ranging from 8 g within the United Kingdom to 20 g within Austria) [34–36]. According to the categories used within the *SHARE* survey, excessive drinking behavior (drinking more than six alcoholic drinks per occasion) was grouped into the following five classes: “Not at all in the last 3 months,” “Less than once a month,” “Once or twice a month,” “Once or up to 4 days a week,” “5 days a week or up to daily.” Observations with “Refusal” or “do not know” were set to missing and excluded from the analysis.

### Statistical analysis

The association between the five personality traits, country, age, years of education, and total household income with number of alcohol drinks was modeled by means of Poisson regression with number of alcohol drinks as the dependent variable in SAS version 9.4 (SAS Institute Inc., Cary, NC). The model was grouped by sex because of apparent interactions of the personality traits with sex.

The association between excessive drinking behavior and the before mentioned variables was estimated by a cumulative logit regression model. The effect of age was presented for a change by 10 years in age for both regression models and expressed as relative risk (RR) or odds ratio (OR) with corresponding 95% confidence intervals (CI). Significance of *p*-values was adjusted for multiple testing by the Bonferroni correction considering that two regression models without any further post-hoc tests were estimated. Therewith, the significance level was set to 2.5%.

## Results

### Descriptive analysis

Of the 76,520 individuals interviewed during the seventh wave of data collection of the *SHARE* survey, 13,950 individuals were asked about their alcohol consumption patterns. After exclusion of cases with missing data on alcohol consumption and/or secondary variables such as the Big Five Inventory (*BFI-10*) data, years of education, or total household income, 13,351 cases were included in the present study. The final study collective consisted of individuals living in 12 different European countries: Austria, Belgium, Czech Republic, Denmark, France, Germany, Greece, Italy, Poland, Spain, Sweden, and Switzerland. The mean age of the included individuals was 72.36 years (standard deviation = 8.35 years), 42.5% (*n* = 5674) were male, and 57.5% (*n* = 7683) were female. Further characteristics of the study sample can be found in Table 1.

**Table 1.** Descriptives of study population (*n* = 13,357).

	% (*n*)	Missing data		
Sex		0		
Male	42.5 (5674)			
Female	57.5 (7683)			
Countries		0		
Austria	3.4 (458)			
Germany	6.1 (813)			
Sweden	7.7 (1024)			
Spain	8.8 (1177)			
Italy	11.1 (1481)			
France	8.3 (1110)			
Denmark	9.4 (1253)			
Greece	13.9 (1859)			
Switzerland	5.5 (740)			
Belgium	11.3 (1512)			
Czech Republic	6.4 (858)			
Poland	8.0 (1072)			
Excessive drinking		4		
Not at all in the last 3 months	79.4 (10,604)			
Less than once a month	9.1 (1215)			
Once or twice a month	5.1 (683)			
Once or up to four days a week	3.7 (497)			
Five days a week or up to daily	2.7 (354)		

	Mean	Lower quartile	Upper quartile	Missing data
Sociodemographic factors				
Age (years)	72.36	66	78	0
Total household income (€)	30,300.00	10,100.00	36,000.00	0
Years of education	10.78	8	14	0
Big 5 personality dimensions				
Openness to new experiences	3.18			0
Conscientiousness	4.09			0
Agreeableness	3.71			0
Extraversion	3.42			0
Neuroticism	2.71			0
Alcohol consumption				
Alcoholic drinks in the last seven days	4.12	0	6	6

### Statistical analysis

#### Influence on the number of alcoholic drinks

Our model revealed the variables country of living (*p* < 0.0001) as well as age (*p* < 0.0001) to exhibit a significant association with alcohol intake for both, males and females—with consumption of alcohol decreasing with increasing age (RR = 0.84; CI = 0.80–0.88 for males and RR = 0.83; CI = 0.80–0.88 for females). Interestingly, also years of education exhibited a significant positive association with alcohol intake for both genders (*p* = 0.0045; RR = 1.01; CI = 1.00–1.02 for males and *p* < 0.0001; RR = 1.04, CI = 1.02–1.06 for females). Our model did not show a significant association with alcohol intake for the variable of total household income. In males, the personality traits conscientiousness, agreeableness, and extraversion were significantly associated with the number of drinks consumed. Extraversion exhibited a significant positive association (*p* < 0.0001) with alcohol intake for males, with higher scores being associated with higher consumption (RR = 1.11; CI = 1.07–1.16). Conversely, the traits conscientiousness (*p* = 0.0012; RR = 0.93; CI = 0.89–0.97) and agreeableness (*p* = 0.0113; RR = 0.94; CI = 0.90–0.99), exhibited a significant negative association with alcohol intake, with higher scores being associated with a lower consumption of alcohol. For the personality traits openness as well as neuroticism, our model did not show any significant influence on alcohol intake for males.

In females, in contrast to data concerning the male collective, the trait openness to new experiences exhibited a significant positive association (*p* = 0.0023; RR = 1.11, CI = 1.04–1.18) with alcohol intake, as higher scores were associated with increased alcohol consumption. For the personality traits conscientiousness, agreeableness, extraversion as well as neuroticism, our model did not show any significant association with alcohol intake in females. All results of the Poisson regression model are displayed in Table 2.

**Table 2.** Relative risk for alcohol consumption (Poisson regression model).

	*Male*		*Female*	
Factor	RR	95% CI	RR	95% CI
Age (10 year groups)	0.84	0.80–0.88	0.83	0.80–0.88
Years of education	1.01	1.00–1.02	1.04	1.02–1.06
Household income	1.00	1.00–1.00	1.00	1.00–1.00
Agreeableness	0.94	0.90–0.99	1.02	0.94–1.11
Conscientiousness	0.93	0.89–0.97	0.97	0.90–1.02
Extraversion	1.11	1.07–1.16	1.04	0.97–1.11
Neuroticism	0.97	0.93–1.01	0.96	0.90–1.02
Openness to new experience	1.00	0.96–1.04	1.11	1.04–1.18

Abbreviations: 95% CI, 95% confidence interval; RR, relative risk.

#### Excessive drinking

Factors associated with excessive drinking behavior (drinking more than six alcoholic drinks on one occasion), were analyzed by a cumulative logit regression model. As gender itself was recognized as influencing factor, the analysis was performed for the whole study sample (and not differentiated by sex) and yielded the following results: An increase in age by 10 years was associated with a significantly (*p* < 0.0001) reduced risk for excessive drinking (OR 0.65; CI 0.62–0.69), being female was associated with a distinct risk reduction for excessive drinking (*p* < 0.0001; OR = 0.34; CI = 0.31–0.37), and also the country of living (*p* < 0.0001) was significantly associated with excessive drinking behavior.

Also, within this model, personality characteristics were associated with the level of excessive drinking behavior: High extraversion was a risk factor for excessive drinking (*p* < 0.0001; OR = 1.15; CI = 1.09–1.21), whereas conscientiousness was shown to act as a protective factor (*p* < 0.0001; OR = 0.87; CI = 0.83–0.93)—see all results of this model in Table 3.

**Table 3.** Odds ratio for excessive drinking (logit regression model).

Factor	OR	95% CI
Age (10 year groups)	0.65	0.62–069
Sex (reference = male)	0.34	0.31–0.37
Years of education	1.01	0.99–1.02
Household income	1.00	1.00–1.00
Agreeableness	0.99	0.93–1.04
Conscientiousness	0.87	0.83–0.93
Extraversion	1.15	1.09–1.21
Neuroticism	1.00	0.95–1.05
Openness to new experience	0.99	0.94–1.04

Abbreviations: 95% CI, 95% confidence interval; OR, odds ratio.

## Discussion

This study aimed to determine risk factors associated with alcohol consumption in older adults by calculating two models, one describing *moderate alcohol consumption* and another one describing *excessive drinking.* Our findings highlight the importance of personality traits as a potential focus for clinicians to identify patients at risk of hazardous alcohol consumption.

Further, the influence of personality traits on drinking patterns differed between the sexes, as extraversion was significantly and positively associated with alcohol consumption for males but not for females. Our results on extraversion as risk factor for increased alcohol intake corroborate previous studies [18,20,37–40]. In a pooled meta-analysis of eight cohort studies, Hakulinen et al. evaluated the influence of the Big Five (*BFI-10*) personality traits on alcohol consumption in more than 70,000 adults [38]. The authors reported an increased likeliness for high-alcohol consumption in individuals exhibiting a high degree of extraversion and a low degree of conscientiousness. In contrast, high levels of agreeableness and low openness to experience were shown to be associated with reduced alcohol consumption or even abstinence. However, some moderators are suggested to significantly alter the influence of personality on alcohol consumption in studies: current treatment for AUD, study design (longitudinal/cross-sectional), and sex [32]. The domain of extraversion subsumes personality traits such as being socially active, gregarious, and excitement-seeking. It is suggested that individuals scoring high at extraversion are more frequently aiming to enhance affective states by increased alcohol use and engage in so-called enhancement-motivated drinking [19,41]. The observed relationship between high degrees of agreeableness and decreased alcohol intake observed within females of our study sample is also in line with previous studies [26,38]. Individuals thinking highly of interpersonal relationships and being received as trust-worthy and compliant (i.e., features of agreeableness) might reduce or limit their alcohol consumption in order to meet expectations of family and close friends [42].

Notably, the present study also revealed conscientiousness to exhibit a significant negative association with alcohol intake for males, which is in line with previous literature [19,43,44]. Indeed, people with high-conscientiousness scores are described as being self-disciplined, acting responsibly, and thoughtful [41] and tend to avoid situations which may adversely impact self-control or their social perception [42].

These observed gender differences of the Big Five personality traits associated with alcohol consumption represent novel findings, as previous studies had either failed to find any sex differences [26,38] or had not tested for them [20,37]. Analyzing personality traits and their possible association with excessive drinking, the model revealed multiple findings of note.

First, no differences in the effects of personality traits on heavy drinking were found between females and males. Extraversion was revealed to be a significant risk factor for excessive drinking, irrespective of the person’s sex. Concurrently, conscientiousness was found to represent a protective factor against excessive drinking. However, males were at significantly higher risk of engaging in excessive drinking than females.

Secondly, our analysis exhibited internal consistency as those personality traits, identified to be a significant factor in the model analyzing *excessive drinking behavior*, showed corresponding effects in the model analyzing *general alcohol intake.* The consistency of this relationship adds further plausibility to the postulated association between certain personality domains and alcohol consumption.

Thirdly, our regression models indicate personality traits to exert sex-specific effects on alcohol consumption in individuals with moderate consumption only. However, for individuals already engaging in excessive drinking, our analyses indicate this behavior to be associated with shared personality traits in both sexes, thus not exhibiting significant sex differences in our analysis.

The depicted relationship of personality traits and alcohol consumption in this multi-national analysis reveals potential implications for the management of *AUD* patients.

Prognosis and relapse risk are described to be substantially affected by personality traits [45,46]. In context with the findings of the present analysis, previous studies suggest knowledge and consideration of personality traits to be highly relevant for treatment of patients with AUD and planning of population-wide preventative measures.

Should tests for personality traits be incorporated in the diagnostic routine, this could be helpful in choosing the most suitable treatment option available, as for psychotherapeutic interventions, maladaptive personality traits are postulated to be significantly associated with treatment outcome [47–49]. Conveniently, screening for personality dimensions is rather feasible and cost-effective: The *BFI-10* questionnaire, which was also used in this analysis, is a self-rating scale and can be completed within a few minutes.

Furthermore, maladaptive personality traits are not only hypothesized to be associated with increased risk for relapse but are also reported to be associated with conditions relevant in the clinical management of patients with *AUD*: decrease in neurocognition, motoric cognitive risk, mild cognitive impairment as well as dementia were found to be associated with personality traits [50,51].

Additionally, our findings indicate that—especially for those patients being on the verge of exhibiting excessive drinking behavior—gender-specific and personality-targeted interventions could be pivotal. However, interpretation of the current findings remains to be confirmed in future research.

Besides personality, socio-economic and socio-demographic factors analyzed in the present study included age, sex, nationality, years of education, and total household income. Not surprisingly, regional differences in alcohol consumption [8] as a consequence of different alcohol consumption policies [8,52], were observed in both models.

Age was significantly negatively associated in both models, corresponding to a decline in older individuals’ alcohol consumption. The variable “years of education” was significantly positively associated with alcohol intake: The longer the educational career, the more alcoholic drinks were consumed. However, this relationship was only valid for the first model (*moderate alcohol consumption*), but not for the second model describing *excessive drinking.* A possible explanation for this association between educational status and alcohol consumption has previously been provided by Li et al. [17]: in western societies, educational status correlates with socioeconomic status and older, higher educated people are more likely to afford alcohol and to engage in social drinking. Furthermore, this association was also reported in a recent survey among more than 5,000 Norwegian adults, where higher education attainment as well as being of a younger age were found to be positively associated with alcohol-related work-impairment [53]. However, the present study population was comprised of individuals aged ≥50 years. Therefore, we cannot assume this negative association of age and alcohol intake to hold true for adolescents or adults younger than 50 years.

Some limitations of the present analysis need to be noted. While the *SHARE* survey employs probability-based sampling [54] to best achieve a representative sample for each country included, not the entire questionnaire was surveyed in all countries. Thus, the number of countries with a complete data set available regarding behavioral risk (i.e., alcohol consumption) and Big Five personality trait questionnaire was limited to 12 out of 27 European countries originally included in the *SHARE* survey. Furthermore, due to the *SHARE* survey’s explicit focus on aging, only participants aged 50 years or older were included, limiting the generalizability of results to the population as a whole. The assessment of personality using the short *BFI-10* contains limitations, as (a) it is not measurement-invariant across all countries [55] and (b) due to time constraints, the short version of the *BFI-10* was used to assess personality—which does not allow a more in depths analysis of the facets that constitute the higher-order personality dimensions.

Notably, methodological heterogeneity in terms of sampling but also in regard to assessing and operationalizing alcohol intake, are generally diminishing the comparability of *AUD/SUD* studies, which is also true for the present study.

These limitations notwithstanding, the authors reiterate that this study, including the current gold standard in personality research, namely the Big Five taxonomy, represents a comprehensive and multi-national evaluation of factors influencing alcohol consumption. Furthermore, potential limitations on availability of socioeconomic data, inherent to the retrospective analysis in the present study, are outweighed by the size of the study population and the professional realization of data collection, including probability-based sampling. Within this multi-national analysis, the relationship between certain socio-economic, socio-demographic factors, and personality traits, in particular extraversion, conscientiousness, and agreeableness, and alcohol intake was confirmed.

## Conclusions

Alcohol consumption in the elderly is affected by socio-economic, socio-demographic factors, and personality characteristics. Country of living, age, and educational history were significant factors in determining alcohol consumption patterns as well as individual personality traits: High levels of extraversion in males and high levels of openness to new experiences in females predict increased alcohol consumption, whereas higher values for conscientiousness and agreeableness were associated with reduced alcohol intake in males. Similarly, hazardous drinking behavior was shown to be affected by age, gender, and country of living but also by personality: Higher levels of extraversion were revealed to be a risk factor for both sexes, whereas conscientiousness was shown to act as a protective factor. These findings suggest that (a) effective policymaking and preventative measures should consider cultural and economic background and that (b) personality assessments should receive more attention in diagnostic and treatment of AUD, especially in terms of prognosis and risk of relapse and for selecting the most feasible type of treatment for every individual patient.

## Data Availability

The data that support the findings of this study are available from SHARE-ERIC. Restrictions apply to the availability of these data, which were used under license for this study. Data are available at www.share-project.org with the permission of SHARE-ERIC.
